# Aerial video & trajectory dataset of vehicles on circular road

**DOI:** 10.1016/j.dib.2025.111858

**Published:** 2025-07-08

**Authors:** Kevin Riehl, Shaimaa K. El-Baklish, Anastasios Kouvelas, Michail A. Makridis

**Affiliations:** Traffic Engineering Group, Institute for Transport Planning and Systems, ETH Zurich, Stefano- Franscini Platz 5 8093 Zurich, Switzerland

**Keywords:** Remote sensing, Vehicle trajectory, Computer vision, Deep learning, Oriented object detection, Intelligent transportation systems

## Abstract

This article presents aerial video and vehicle trajectory data collected during a phantom traffic jam experiment with the Swiss television (SRF) at the driving test centre TCS Derendingen, Solothurn, from March 12th 2024. 14 vehicles were recorded for a total duration of 40 minutes with a drone from above, and vehicle trajectories were extracted using computer vision and Kalman filtering methodology. The observed vehicles differ by their power train (combustion, electric, hybrid), gearbox (manual, automatic), and equipment with advanced driver assistance systems.

The data provided in this article offers a valuable resource for researchers, industry representatives, public authorities, and other parties interested in mixed-traffic dynamics, traffic flow theory, computer vision. This dataset can for instance be used: (i) to explore how gearbox, powertrain, and assistance systems affect the propagation of traffic jams on highways, and (ii) to provide a benchmark dataset for visual vehicle trajectory extraction using computer vision methods.

Specifications Table

**Table:** 

Subject	Computer Sciences
Specific subject area	*Vehicle trajectories and powertrain dynamics in a phantom traffic-jam, ring-road setup*
Type of data	*Video Files (.mov format), Raw Object Annotations (.csv format), Processed Vehicle Trajectories (.csv format)*
Data collection	*The aerial videos were recorded during an experiment with 14 vehicles of different size, colour, and shape, that were driving on a circular test road, from a height of around 50m. For video recording, drone model DJI AIR 2S was used. The video material has a resolution of 3840×2160 pixels, a rate of 25 frames per second, and includes 40 minutes of recording (50 GB of data). The trajectories were extracted from videos using object detection and Kalman filtering.*
Data source location	*The videos were recorded on March 12th 2024, in cooperation with Laurin Merz, Hook-Film, and Adrian Winkler and Andrea Fischli, Swiss Television Schweizer Radio und Fernsehen (SRF, tv-show Einstein from May 2nd 2024), at the TCS Driver Training Centre in Derendingen, Solothurn (Switzerland, 47.195458894398506, 7.597843483113585). The planning of the experiment was conducted by Kevin Riehl, and the execution was supported by Andre Greif.* https://www.srf.ch/play/tv/einstein/video/stau-was-hilft-gegen-den-verkehrskollaps?urn=urn:srf:video:63965781-c7ea-4033-9827-be4275f1cba5
Data accessibility	Repository name: Zenodo Data identification number: 10.5281/zenodo.15124431 Direct URL to data: https://doi.org/10.5281/zenodo.15124431 Repository name: GitHub Data identification number: https://github.com/DerKevinRiehl/trajectory_analysis Direct URL to data: https://github.com/DerKevinRiehl/trajectory_analysis Repository name: Youtube Data identification number: https://www.youtube.com/watch?v=R8mvTePXp6M Direct URL to data: https://www.youtube.com/watch?v=R8mvTePXp6M
Related research article	*Consistent Vehicle Trajectory Extraction From Aerial Recordings Using Oriented Object Detection (Currently in Submission with Scientific Reports)*

## 1 Value of the Data

• **Unique insights into traffic jam dynamics:** The dataset provides a view of how phantom traffic jams develop and propagate in a closed-loop controlled environment. By offering vehicle trajectories captured with high precision through drone footage and advanced computer vision techniques, this dataset enables the study of traffic phenomena in a real-world context.
• **Heterogeneous, mixed-traffic vehicle analysis:** The data captures various vehicle attributes, such as size, power-train type (combustion, electric, hybrid), gearbox type (manual, automatic), and equipment with advanced driver assistance systems (adaptive cruise control). This heterogeneity allows researchers to explore the impact of these factors on traffic behaviour, making it applicable for studies on vehicle dynamics, road safety, traffic jams, and energy consumption in different traffic conditions.
• **Benchmark for computer vision and trajectory extraction:** The dataset serves as a valuable benchmark for testing and improving computer vision algorithms, specifically for vehicle detection and trajectory extraction. Researchers working on artificial intelligence, machine learning, or computer vision applications in traffic analysis will find this dataset useful for validating new techniques.
• **Interdisciplinary applications:** The data is not limited to traffic researchers but also holds value for the automotive industry, transportation planners, and environmental scientists. It enables cross-disciplinary studies on how technological factors such as power-trains, assistance systems, and gearboxes influence overall traffic behaviour. Moreover, autonomous driving applications could be enhanced.

## 2 Background

Vehicle trajectories offer valuable insights for a wide range of road transportation applications and research fields. Trajectories allow to model realistic driving behaviours, such as car-following & lane-changing, and real-world traffic patterns, such as oscillation propagation & capacity drops. In addition to that, trajectories are helpful when developing driving safety systems and support road infrastructure planning, design and control [[7], [8]].

This dataset provides novel insights for the domains of traffic flow theory, traffic modelling and simulation, vehicle powertrain dynamics, energy consumption, and emissions [[3], [4], [5]]. Capturing vehicle dynamics in a controlled environment allows for a unique analysis of how traffic jams develop and propagate when different gearbox, assistance systems, and power-trains are present [6], providing rare insights into inter-vehicle dynamics and congestion propagation on highways.

Besides, this dataset contributes to the emerging field of computer vision and remote sensing in transportation, and supports development of more accurate and robust computer vision methods for vehicle detection and trajectory extraction in real-world traffic scenarios. This dataset serves as a benchmark for improving and validating computer vision algorithms, particularly for those focussed on vehicle detection and trajectory extraction.

Beyond traffic research, this dataset also has interdisciplinary applications, supporting studies in the automotive industry, transportation planning, and environmental science, particularly in areas such as road safety, energy consumption, and autonomous driving [9].

**Fig. 1:**
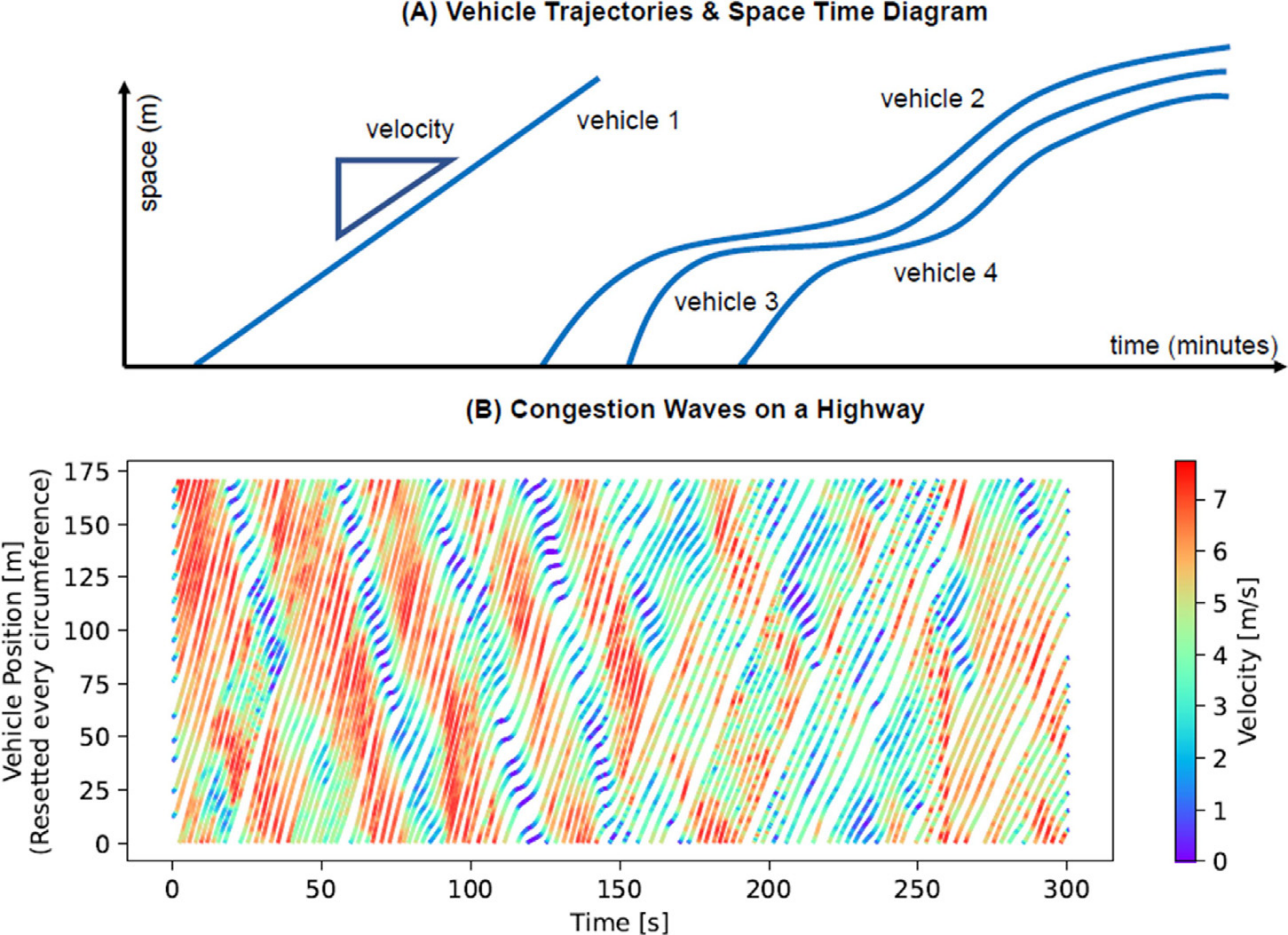
Vehicle trajectories displayed in space time diagrams. (A) Four vehicles are shown. The slope of each graph represents the vehicles velocity. Vehicle 1 drives at a constant speed. Vehicles 2, 3, and 4 get closer to each other over time during braking manoeuvres (e.g. for a red traffic light), and afterwards follow each other closely with small distances. (B) Time-space diagram for the circular track and fifteen observed vehicles in the dataset. The colours represent different levels of speed and reveal stop-and-go waves.

Fig. 1 shows an excerpt of the time-space diagram of 15 vehicles driving on the circular track. Significant stop-and-go waves are evident and string instability can be studied.

Although there are some datasets with human driving vehicles and partially-automated vehicles in the literature, there is scarcity on datasets involving mixed traffic flow and available vehicle specifications. The closed-loop experiment on a ring-road offers a unique playground to study analytically driver behaviour characteristics and vehicle dynamics in the context of vehicle properties.

## 3 Data Description

**Fig. 2:**
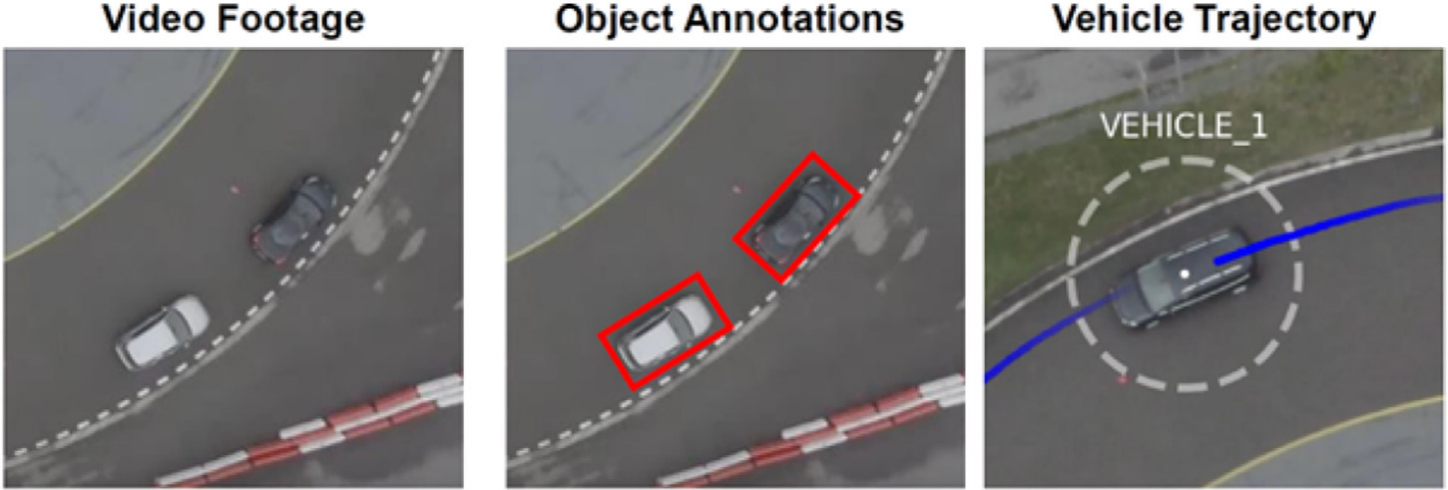
Components of the dataset.

This dataset consists of three components: (i) video recordings, (ii) object annotations, and (iii) vehicle trajectories, as shown in Fig. 2.

The fifteen **video recordings** (see Table 1) cover a duration of around one hour (01:02:56) and 94,423 frames in total, at a frame-rate of 25 frames per second, and with a resolution of 3840×2160 pixels. The videos show more than 14 vehicles driving on the ring road of the driving test centre TCS Derendingen (Solothurn, Switzerland) during various experiments. The videos are provided in MOV format and can be downloaded from here: https://doi.org/10.5281/zenodo.15124431.

**Table 1:** Experiments & video recordings.

Experiment	Video	Frames	ADAS	Vehicles	Lane	Description
1	DJI 0933.MOV	7518	inactive	14	outer	14 Vehicles, lower density due to outer circle, without ADAS, varying speeds, observing stop-and-go waves
	DJI 0934.MOV	7518	inactive	14	outer	
2	DJI 0939.MOV	7519	active	14	inner	14 Vehicles, higher density due to inner circle, with ADAS, observing fewer congestion
	DJI 0940.MOV	7172	active	14	inner	
3	DJI 0943.MOV	7518	active	15	inner	15 Vehicles, higher density due to inner circle, without ADAS, observing more congestion
	DJI 0944.MOV	7518	active	15	inner	
None	DJI 0931.MOV	7518	inactive	14	-	(driving in uncontrolled experiment)
	DJI 0932.MOV	7519	inactive	14	-	
	DJI 0935.MOV	3535	inactive	14	-	
	DJI 0936.MOV	7518	inactive	14	-	
	DJI 0937.MOV	3675	inactive	14	-	
	DJI 0938.MOV	7518	inactive	14	-	
	DJI 0941.MOV	694	inactive	14	-	
	DJI 0942.MOV	7518	inactive	15	-	
	DJI 0945.MOV	4165	inactive	15	-	

The **object annotations** provide for each video and frame a list of rectangular annotations that envelop a vehicle, generated by 18 different object detection models. The annotations are provided as zipped CSV files, separated by the tabulator symbol. The parameters provided in such files are shown in Table 2. The annotation category follows the DOTA dataset classification system [10]; for example, a category ID of 10 stands for a small vehicle. The object annotations can be downloaded from here: https://doi.org/10.5281/zenodo.15124431.

**Table 2:** Parameters of the object annotations extracted from the video footage by the oriented object detection model.

Parameter	Description	Example Value	Unit
Frame	Numeric frame ID (i.e. number)	76	[-]
Category	Numeric classification ID	10	[-]
X	Bounding box center	2120.2	[px]
Y	Bounding box center	1958.6	[px]
Width	Bounding box width	129.1	[px]
Height	Bounding box height	63.5	[px]
Angle	Angle (i.e. orientation) of the bounding box	0.0673	[rad]
Confidence	Confidence of annotation; between 0 and 1	0.0562	[-]

**Fig. 3:**
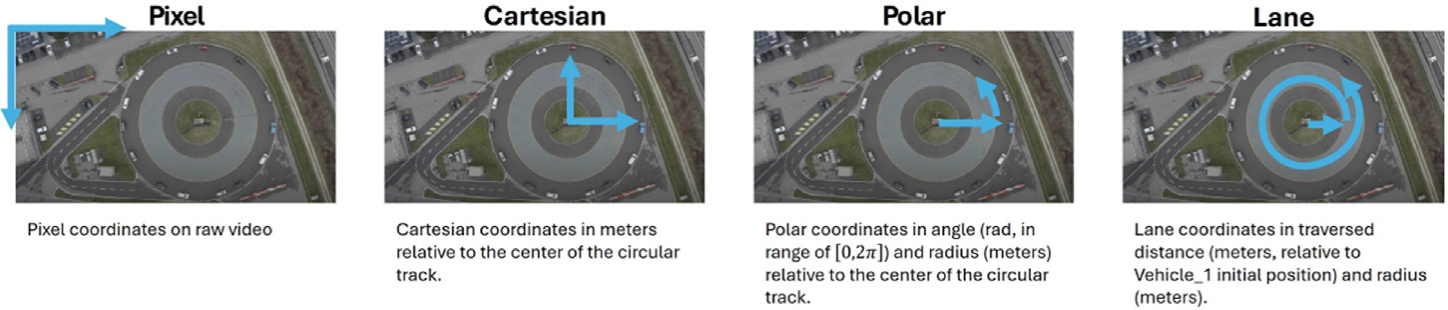
Coordinate Systems. The coordinates of detected vehicles have been derived using object detection algorithms in pixel coordinates (far left) and then converted to other coordinate systems until reaching the lane coordinates (far right).

The **vehicle trajectories** provide for each video, vehicle, and frame an exact vehicle position. The vehicle trajectories are provided as zipped CSV files, separated by the comma symbol. The parameters provided in such files are shown in Table 3. The coordinate systems used are illustrated in Fig. 3. The coordinates in Cartesian coordinates are in reference to the centre of the circle road in the driving test centre TCS Derendingen (47.19515354751697, 7.599108486109998). The field Lane X Ref reflects the lane coordinate assuming that Vehicle 1’s position in the video’s first frame is considered as starting point (0 m). The polar coordinates and lane coordinates are provided only for the videos that were related to the execution of experiments (DJI 0933.MOV, DJI 0934.MOV, DJI 0939.MOV, DJI 0940.MOV, DJI 0943.MOV, DJI 0944.MOV). The vehicle trajectories can be downloaded from here: https://doi.org/10.5281/zenodo.15124431.

**Table 3:** Parameters of the vehicles’ trajectory data extracted from the video footage.

Parameter	Description	Example Value	Unit
Vehicle_ID	String ID of the vehicle	VEHICLE_1	[-]
Frame_ID	Numeric frame ID (i.e. number)	3	[-]
Global_Time	Time from the start of the video	0.12	[s]
Cartesian	[Cartesian_X, Cartesian_Y]	[-16.48, 25.65]	[m], [m]
Polar	[Polar_X, Polar_Y]	[4.14, 30.49]	[rad], [m]
Lane	[Lane_X, Lane_Y]	[128.83, 30.49]	[m], [m]
Dimension	[v_Length, v_Width]	[4.36, 1.51]	[m], [m]
v_Vel	Longitudinal speed of the vehicle	1.85	[m]
v_Angle	Orientation of the vehicle	5.69	[rad]
v_AngleVel	Angular velocity of the vehicle	0.29	[rad/s]
Proceeding	String ID of the proceeding vehicle	VEHICLE_14	[-]
Headways	[Space_Hdwy, Time_Hdwy]	[9.71, 5.24]	[m], [s]
v_Accel	Longitudinal acceleration of the vehicle	2.37	[m/s^2^]

### 3.1 Experimental design, materials and methods

Three experiments at the driver test centre Derendingen were conducted, varying different number of vehicles, varying activation of advanced driver assistance systems (ADAS), and ring road length, to analyse the effects of different vehicle densities and traffic congestion. The experiment details are outlined in Table 4. The vehicle details, including colour, position, power-train, gearbox, ADAS equipment, and car model are outlined in Table 4. The vehicles were provided by a rental car company related to the driving test centre to ensure absence of sensitive private data and license plate information.

**Table 4:** Vehicle information table.

Nr.	Color	Pos.	Brand	Model	Year	Powertrain	Gearbox	ADAS	Displ. [l]	Power [HP]	Torque [Nm]
1	Black	[1]	SEAT	Alhambra II	2022	Combustion	Automatic	Yes	2.0	150	340
2	Gray	[2]	Toyota	BZ 4 X	2022	Electric	Automatic	Yes	-	218	266
3	Dark gray	[3]	Renault	Laguna III	2015	Combustion	Manual	No	2.0	150	340
4	Silver	[4]	VW	Scharan TSI	2017	Combustion	Automatic	No	1.4	150	250
5	Silver	[5]	Renault	Laguna III	2014	Combustion	Automatic	No	2.0	150	340
6	Gray	[6]	Toyota	Verso D-4D	2015	Combustion	Automatic	No	2.2	177	400
7	Red	[7]	Citroen	Picasso C3 VTi 120 EGS6	2015	Combustion	Manual	No	1.6	120	160
8	Black	[8]	Ford	Focus NA3	2018	Combustion	Manual	No	1.5	150	240
9	Beige	[9]	VW	Caddy Maxi TSI	2019	Combustion	Automatic	Yes	1.4	125	200
10	Light blue	[10]	Volvo	Polestar 1, Race version	2021	Combustion	Automatic	Yes	2.0	600	1000
11	White	[11]	VW	Caddy 2 K TSI	2012	Combustion	Manual	No	1.2	86	160
12	Black	[12]	Peugeot	RCZ R	2015	Combustion	Manual	No	1.6	270	330
13	Gray	[13]	VW	Golf, Gold VII Type AU	2014	Electric	Automatic	Yes	-	115	270
14	White	[14]	Audi	A4 TDI DSG	2016	Combustion	Automatic	Yes	2.0	190	400

**Additionally inserted in experiment 3:**											
15	Black	[after 2]	Opel	Adam 1.4	2019	Combustion	Manual	No	1.4	87	130

The videos were recorded using drone model DJI AIR 2S from 50 meters above the ground. The object annotations were computed using oriented bounding box object detection algorithms based on convolutional neural network. The model s2anet r50 fpn fp16 1x dota le135 [1,2] was chosen for this purpose, as it accurately outperforms in terms of detection performance; details on implementation can be found here https://github.com/open-mmlab/mmrotate/blob/main/configs/s2anet/README.md.

The vehicle trajectories were computed using a conditional, time-discrete, non-linear, Extended Kalman Filter [11,12] to complete gaps in the trajectories, to reduce noise in positional and angular information, and to improve trajectory quality overall. An online video (YouTube) on the attached GitHub repository demonstrates the functioning of the vehicle trajectory methodology.

## Limitations

‘None’ or ‘Not applicable’.

## Ethics Statement

The authors confirm that the they have read and follow the ethical requirements for publication in Data in Brief and confirming that the current work does not involve human subjects, animal experiments, or any data collected from social media platforms.

## CRediT authorship contribution statement

**Kevin Riehl:** Conceptualization, Methodology, Software, Validation, Formal analysis, Investigation, Resources, Data curation, Writing – original draft, Visualization, Project administration. **Shaimaa K. El-Baklish:** Conceptualization, Methodology, Software, Validation, Formal analysis, Investigation, Data curation, Writing – review & editing, Visualization. **Anastasios Kouvelas:** Writing – review & editing, Supervision. **Michail A. Makridis:** Writing – review & editing, Supervision.

## Data Availability

YouTubehttps://www.youtube.com/watch?v=R8mvTePXp6M (Original data). GitHubhttps://github.com/DerKevinRiehl/trajectory_analysis (Original data). Zenodohttps://doi.org/10.5281/zenodo.15124431 (Original data).
